# Best Practice Guide for Reducing Barriers to Video Call–Based Telehealth: Modified Delphi Study Among Health Care Professionals

**DOI:** 10.2196/64079

**Published:** 2025-03-26

**Authors:** Lena Rettinger, Lea Aichinger, Veronika Ertelt-Bach, Andreas Huber, Susanne Maria Javorszky, Lukas Maul, Peter Putz, Sevan Sargis, Franz Werner, Klaus Widhalm, Sebastian Kuhn

**Affiliations:** 1 Research Center Digital Health and Care FH Campus Wien, University of Applied Sciences Vienna Vienna Austria; 2 Institute of Digital Medicine Philipps-University & University Hospital of Giessen and Marburg Marburg Germany; 3 Occupational Therapy FH Campus Wien, University of Applied Sciences Vienna Vienna Austria; 4 Orthoptics FH Campus Wien, University of Applied Sciences Vienna Vienna Austria; 5 Logopedics – Phoniatrics – Audiology FH Campus Wien, University of Applied Sciences Vienna Vienna Austria; 6 Research Center Health Sciences FH Campus Wien, University of Applied Sciences Vienna Vienna Austria; 7 Midwifery FH Campus Wien, University of Applied Sciences Vienna Vienna Austria; 8 Physiotherapy FH Campus Wien, University of Applied Sciences Vienna Vienna Austria

**Keywords:** telehealth, best practices, video call, Delphi study, health communication, barriers, health care professionals, qualitative interviews, web-based survey, physiotherapists, speech therapists, language therapists, dietitians, midwife

## Abstract

**Background:**

Telehealth has grown, especially during the COVID-19 pandemic, improving access for those in remote or underserved areas. However, its implementation faces technological, practical, and interpersonal barriers.

**Objective:**

The aim of this study was to identify and consolidate best practices for telehealth delivery, specifically for video call sessions, by synthesizing the insights of health care professionals across various disciplines.

**Methods:**

We first identified 15 common telehealth barriers from a preceding scoping review. Subsequently, a modified Delphi method was used, involving 9 health care professionals (physiotherapists, speech and language therapists, dietitians, and midwife) with telehealth experience in qualitative interviews and 2 iterative rounds of web-based surveys to form consensus.

**Results:**

This study addressed 15 telehealth barriers and identified 105 best practices. Among these, 20 are technology-related and 85 concern health care practices. Emphasis was placed on setting up telehealth environments, ensuring safety, building relationships and trust, using nonmanual methods, and enhancing observation and assessment skills. Best practice recommendations for dealing with patients or caregiver skepticism or lack of telehealth-specific knowledge were developed. Further, approaches for unstable networks and privacy and IT security issues were identified. Areas with fewer best practices were the lack of technology skills or technology access, unreliability of hardware and software, increased workload, and a lack of caregiver support.

**Conclusions:**

This guide of best practices serves as an actionable resource for health care providers to navigate the complexities of telehealth. Despite a small participant sample and the potential for profession-specific biases, the findings provide a foundation for improving telehealth services and inform future research for its application and education.

## Introduction

Video call–based telehealth refers to the use of information and communication technologies to deliver health care services remotely and synchronously, bridging the gap between patients and health care providers [1]. Over the past few years, telehealth has seen an increase in adoption due to its potential to improve access to health care services, particularly for individuals living in rural or remote areas [2]. The COVID-19 pandemic has further accelerated this trend, as the need for remote health care solutions to reduce the risk of infection transmission has become more urgent [3]. As a result, telehealth has emerged as an essential part of modern health care delivery, offering new opportunities and challenges for both patients and health care providers.

Telehealth has the potential to offer numerous advantages and benefits for patients, health care providers, and the overall health care system. Benefits summarized in previous reviews include, for example, increased access and convenience, cost savings, improved family involvement, and better management [4]. Increased access refers particularly to individuals living in remote or underserved areas [2], as well as to those with mobility limitations or chronic conditions that require frequent monitoring [5]. Furthermore, telehealth enables patients to receive care from the comfort of their own homes, reducing the need for travel and waiting times associated with in-person appointments and providing a more convenient way of service provision [6]. By reducing travel costs, decreasing hospital readmissions, and minimizing the need for in-person consultations, telehealth might also have the potential to reduce health care costs for both patients and providers [7,8].

Despite its numerous advantages, telehealth also faces several challenges and barriers that can hinder its successful implementation. Recent studies and reviews have highlighted various obstacles to delivering care via real-time video consultations. These include technical issues such as unstable internet connectivity [4,9,10], lack of technology skills among both patients and providers [4,9,11], and concerns over privacy and data security [9,12]. Moreover, health care professionals have reported challenges in establishing rapport and trust with patients via video call [9,11], and there exists skepticism about the effectiveness of remote care compared to in-person sessions [9]. Addressing these barriers is especially critical when considering the rapid shift to telehealth prompted by the COVID-19 pandemic, which has underscored the potential benefits of video consultations but also exposed the need for clearer guidelines to ensure quality of care [9,13]. Despite growing evidence on these challenges, the literature lacks a comprehensive set of actionable best practices to mitigate barriers associated with video consultations. This study therefore aims to fill that gap by drawing on multiple health care disciplines and using a modified Delphi process to achieve expert consensus on best practices to maximize the potential benefits of video call–based telehealth for patients, health care providers, and the overall health care system [14].

## Methods

### Overview

In this study, we used a modified Delphi method [15] to identify best practices to overcome common barriers in video call–based telehealth. The reporting follows the proposed Delphi reporting guideline by Spranger et al [16].

### Research Paradigm

The epistemological positioning of this study is based on a pragmatic paradigm that recognizes the value of diverse viewpoints and the importance of practical solutions to real-world problems. Pragmatism has been identified as relevant and useful for qualitative research on organizational processes [17], as well as in patient-oriented research [18]. This approach also aligns with the goals of the modified Delphi method, which aims to synthesize expert knowledge into actionable best practices. It acknowledges the dynamic and iterative nature of knowledge creation and embraces subjective experiences to address the complex challenges of telehealth implementation.

### Study Design

Our modified Delphi method was characterized by 2 main adaptations to the traditional Delphi process. First, we used preliminary expert interviews to gather an initial pool of best practices. These interviews guided the development of a list of 131 practice statements and 15 barriers, which were then presented to the expert panel for rating. Second, our survey structure diverged from a classic Delphi by using (1) a 4-point Likert scale and (2) an additional response option “This practice is not relevant for my profession.” This allowed professionals from various health care disciplines to opt out of rating statements they found inapplicable. We also set specific thresholds for acceptance (≥80% agreement after round 1; ≥75% agreement after round 2) to ensure each item reached a robust consensus without necessitating a third round. These modifications provided a more streamlined approach and helped account for the diverse professional backgrounds of the participants and time and resource limitations.

### Recruitment

We recruited participants via a combination of email invitations, professional social media groups, and a snowball sampling strategy. Known telehealth practitioners were first contacted directly by the research team and then invited to share the invitation within their professional networks. To ensure broad representation across allied health professions, we distributed the study invitation to professional associations and specialized digital communities. To be eligible, participants had to (1) have at least 2 years of professional experience in a non-physician health care field (eg, nursing, dietetics, occupational therapy, speech-language therapy, physiotherapy, orthoptics, or midwifery), (2) have delivered a minimum of 10 video call–based telehealth sessions in the preceding quarter, and (3) have excellent German language proficiency. All participants were given detailed information about the study objectives, procedures, and time commitments.

### Participants

A total of 9 health care professionals (4 physiotherapists (PTs), 3 speech and language therapists (SLTs), 1 dietitian, and 1 midwife) were purposively recruited based on their experience with telehealth. All participants were licensed and currently practicing health care professionals with at least 2 years of experience in their field. They each had conducted a minimum of 10 video-based telehealth sessions in the preceding quarter and practiced in an area where telehealth is applicable. Their professional experience ranged from 2 to 32 years, and encompassed diverse health care settings such as neurorehabilitation, musculoskeletal care, adults, and pediatric care, bariatric medicine, mixed practice settings, respiratory therapy, independent midwifery, and pelvic health. In total, 4 panelists were female and 5 were male.

### Interviews

The expert interviews were conducted using Zoom videoconferencing software (Zoom Video Communications) in September 2022. Specifically, 4 interview sessions took place: 3 small-group focus groups and 1 individual interview. Focus group 1 included 2 PTs and lasted 1 hour 34 minutes; focus group 2 included 2 SLTs and lasted 1 hour 3 minutes; and focus group 3 included 2 PTs, 1 SLT, and 1 dietitian, lasting 1 hour and 32 minutes. The one-on-one interview was conducted with a midwife and lasted 29 minutes. We used a semistructured interview guide to help focus the discussions, which was based on commonly reported barriers with video call–based telehealth, found in the existing research for these health care fields (Multimedia Appendix 1). All interviews were conducted by the lead author (LR), who is experienced in qualitative research methods and trained in facilitating interviews and focus groups.

The barriers discussed during the expert interviews were identified through a preceding scoping review [9]: Lack of technology access, unstable internet connections, lack of privacy & IT security, lack of technology skills, unreliability of hardware and software, reluctance of patients or caregivers to use telehealth, decreased establishment of relationships and trust, decreased validity of observations and assessments, lack of hands-on methods, decreased quality and effectiveness, lack of telehealth training, increased workload, lack of support for patients from caregivers, increased safety risks for patients, and unsuitability of setting for telehealth. Experts were asked to provide insights and best practices for addressing these barriers, based on their experience.

Each interview was transcribed verbatim by the lead author (LR) with support from an automated transcription software. After automated transcription, LR reviewed the transcripts to ensure accuracy and fidelity to the original recordings. The final transcripts were analyzed using qualitative content analysis, following the framework proposed by Kuckartz and Rädiker [19]. The first author (LR) conducted the initial deductive coding by extracting passages that contained strategies for overcoming telehealth barriers. These excerpts were then assigned to the corresponding barriers. For each identified strategy, the main characteristics and essential elements were extracted to form a clear and actionable statement. To enhance the reliability of the coding, the research team engaged in multiple team rounds to discuss the initial findings. During these sessions, the team identified similarities across different strategies, combined overlapping or related strategies to reduce redundancy, and formulated precise statements that accurately captured the essence of each strategy. Through iterative discussions, codes were refined and adjusted to better represent the data, thereby enhancing the credibility of the findings.

### Ethical Considerations

Prior to participation, all individuals provided informed consent, thereby ensuring ethical compliance and voluntary engagement in the study. The ethics committee confirmed the adherence to ethical principles, and an ethical approval was not required as the study involved only expert consultations without collecting or processing sensitive patient data. All participants gave informed consent, and data collection did not involve patient data. The Ethics Committee for Research Activities of FH Campus Wien, University of Applied Sciences, confirmed that no formal approval was needed for this study (EK 243/2025). The participants did not receive any financial compensation.

### Delphi Rounds

Following the qualitative interviews, we conducted Delphi rounds to achieve expert consensus on best practices for video call–based telehealth. The Delphi process was carried out in 2 rounds. Round 1 was initiated at the beginning of November 2022 and remained open for 2 weeks. During this round, panelists received a web-based survey containing a demographic data section, including name and profession, and 15 sections on barriers and corresponding strategies. Round 2 was conducted at the end of November and beginning of December 2022, also open for 2 weeks. In this round, panelists were presented with a survey of 11 sections with the remaining best practices that were lacking consensus.

#### Barriers

Barriers were rated for their perceived magnitude (1=very small barrier, 2=rather small barrier, 3=rather big barrier, 4=very big barrier) in round 1. Frequency distributions were calculated to determine the perceived magnitude of each barrier among panelists. These results serve to contextualize the forthcoming best practice recommendations.

#### Analysis of Best Practices

Best practices, on the other hand, were rated for their perceived helpfulness (1=not helpful, 2=rather not helpful, 3=rather helpful, 4=very helpful). In recognition of the diverse professional backgrounds of the panelists, the survey items included an additional response option: “This practice is not relevant for my profession.” Additionally, the survey invited the panelists to contribute further comments and propose new best practices not previously covered, thereby contributing to a more comprehensive dataset.

Response percentages were calculated, and the perceived helpfulness of each practice was quantified using frequency distributions for each response category. Following the first survey round, any practice that received a “helpful” or “very helpful” rating from ≥80% of participants and elicited no comments was automatically included in the final guide. Practices with ratings between 50% and 79% or those that received substantive comments moved on to the second round for reevaluation. Regarding practices rated “helpful” or “very helpful” by <50% of participants, we weighed the risk of prematurely discarding potentially relevant items against the need to maintain focus on practices demonstrating at least a minimal level of acceptance by the panel. After careful consideration, the study team decided to exclude these practices immediately following round 1, rather than carrying them forward. However, participants were given opportunities to propose modifications or new practices at the end of each round, ensuring that any critical practice could be reintroduced or reframed if panelists believed it had merit. The research team discussed all received comments and considered if any rephrasing based on the comments should be made. In case the comments led to a rephrasing of a best practice, both the original and revised versions were presented in the second round for further evaluation. During this round, panelists were provided with the remaining best practices along with the percentage of previous agreement and anonymized comments for each best practice in December 2022. Participants were offered 2 response options: “This practice should be included in the best practice guide” or “This practice should not be included in the best practice guide.” When 2 variations of a practice were presented, panelists could select their preferred version or “none of both.” Practices that carried over from round 1 required at least 75% agreement among participants to be included in the final best practice guide [20]. This slightly lower threshold accounted for the fact that these practices already showed moderate support or warranted further discussion due to comments. In case 2 versions of a best practice existed, the version receiving the higher preference was selected.

## Results

### Barriers and Best Practices

**Figure 1:**
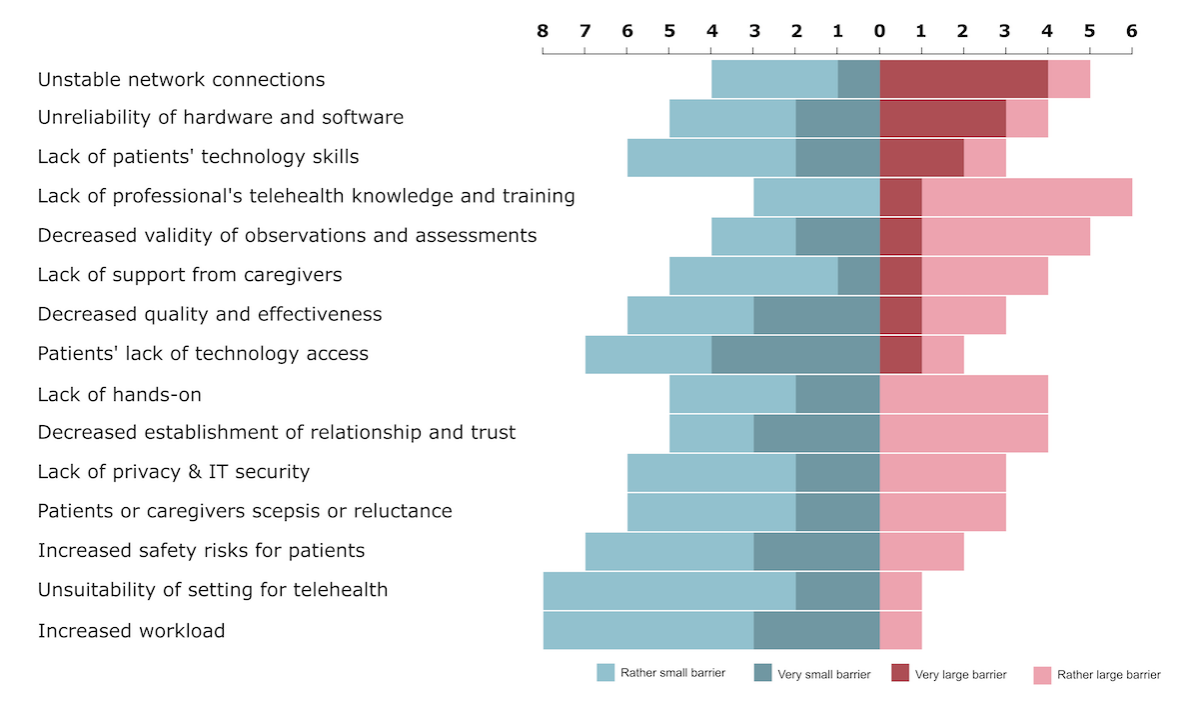
Panelists’ (n=9) perceived magnitude of the 15 barriers presented (bars represent absolute frequencies).

The qualitative content analysis on the expert interviews yielded a total of 422 coded text segments. From this process, an initial set of 131 practices was developed. The association of the practices with the discussed barriers can be found in Table 1, and the perceived importance of the 15 presented barriers is shown in Figure 1.

**Figure 2:**
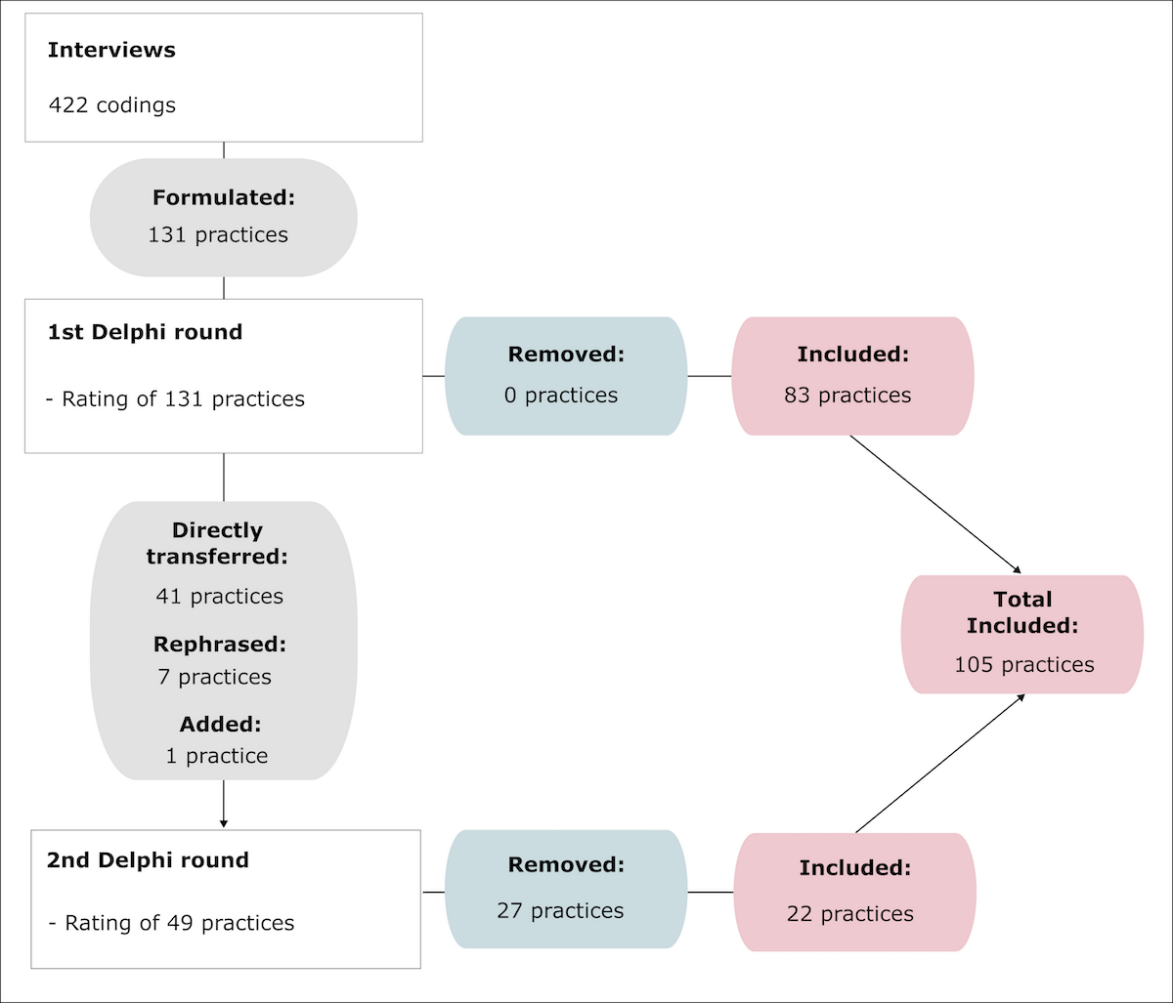
Flowchart of the Delphi process.

In the first Delphi round, 108 practices exceeded the 80% approval threshold for perceived helpfulness; 83 of these practices had no comments and were therefore included directly. In total, 48 practices met the criteria to proceed to the second round: 23 practices rated as “very helpful” or “helpful” by 50%-79% of the experts; 18 without changes but with comments from the first round; and 7 practices with alternative revision based on comments. One entirely new strategy was introduced (Figure 2). Upon completion of the second round, the final best practice guide emerged with 105 practices, covering practices addressing 5 technology barriers and 10 practical barriers (Table 2 and Textbox 1).

In total, we identified 105 best practices addressing 15 telehealth barriers, with 20 aimed at technology challenges and 85 focused on practice issues.

**Table 1:** Demographic characteristics of the panelists.

Panelist number	Gender	Profession	Years of professional experience	Professional experience
1	Male	Physiotherapist	15	Orthopedics, traumatology, neurolrehabilitation
2	Male	Physiotherapist	3	Orthopedics, musculoskeletal, training
3	Female	Speech and language therapist	20	Swallowing disorders, functional voice disorders
4	Female	Speech and language therapist	2	Childhood speech development disorders
5	Male	Physiotherapist	32	Orthopedics, respiratory therapy
6	Female	Dietitian	4	Bariatrics
7	Male	Speech therapist	2.5	General speech therapy, respiratory therapy
8	Female	Physiotherapist	20	Pelvic floor therapy
9	Female	Midwife	28	Birth preparation, support, and aftercare

**Table 2:** Number of best practices for each presented barrier.

Barrier		Number of practices after expert interviews	Number of practices, included after round 1	Number of practices included after round 2	Final number of practices
**Technology barriers**					
	Unstable network connections	9	3	3	6
	Unreliability of hardware and software	4	1	1	2
	Lack of technology skills	6	2	2	4
	Lack of privacy and IT security	7	2	3	5
	Lack of technology access	3	0	3	3
**Health care practice barriers**					
	Lack of professional’s telehealth knowledge and training	8	6	0	6
	Decreased validity of observations and assessments	13	8	2	10
	Lack of support from caregivers	3	1	0	1
	Lack of hands-on	11	7	3	10
	Reluctance of patients or caregivers to use telehealth	8	5	2	7
	Decreased establishment of relationship and trust	11	9	1	10
	Decreased quality and effectiveness	10	9	1	10
	Increased safety risks for patients	13	10	1	11
	Unsuitability of setting for telehealth	21	18	0	18
	Increased workload	4	2	0	2
	Sum total	131	83	22	105

**Best practice guide for barriers to telehealth via video call.**

**Best practices to address technology barriers**

**A. Unstable network connections**

1. Every participant should choose a location with a stable network connection for video call sessions. This should be communicated to the clients in advance.
2. Other devices, persons, or activities should not use the same network connection during the video call session.
3. In case of an unstable network connection, the device may be exchanged.
4. In case of an unstable network connection, switching off the camera for a short time could help.
5. In case of network disconnection, the client should be informed about further steps by telephone (full agreement from all participants).
6. In case of audio transmission problems, an additional phone connection can be used.

**B. Unreliability of hardware and software**

1. The video call software used should be installed and tested in a timely manner by all participants before the start of the session.
2. If clients experience technical issues, possible solutions can be discussed and solved directly during the video call session or via telephone (full agreement from all participants).

**C. Lack of technology skills**

1. Health care professionals should gain digital literacy skills in using different devices and video call software, as this can help in solving common technical problems encountered by patients (full agreement from all participants).
2. For technically non-proficient individuals, user-friendly video call software should be selected. Getting started via a direct link without prior installation should be favored (full agreement from all participants).
3. Telephone support can help inexperienced individuals get started with video call software (full agreement from all participants).
4. As required, the operation of the video call software can be supported at the beginning or ongoing by relatives or other persons on site.

**D. Lack of privacy & IT security**

1. Software used should ensure encrypted data transmission (full agreement from all participants).
2. One's own decisions regarding the handling of client data should be made and communicated in accordance with current data protection laws.
3. Written consent from clients should be obtained in advance.
4. When using video call software, access should only be possible for invited individuals (full agreement from all participants).
5. If software has not been recommended by a certification body for use in health care, it should be reviewed concerning its data processing.

**E. Lack of technology access**

1. Providing a checklist of minimum technical requirements for video call sessions can optimize conditions in advance.
2. Availability of equipment should be confirmed prior to the beginning of the session.
3. For sessions that focus on visual demonstration or perception, it is recommended to use a device with a large, high image quality screen.

**F. Lack of telehealth knowledge and training**

1. Watching video call software tutorials can provide information on available functions and give confidence in handling.
2. Learning by doing can help to gain confidence in conducting video call sessions.
3. A test run with individuals from the private environment can provide confidence in using the technology (full agreement from all participants).
4. Materials for use in video call sessions can be researched on the internet (full agreement from all participants).
5. Reading current articles on the topic of telehealth can increase knowledge (full agreement from all participants).
6. Exchanging experiences with colleagues about the use of video units can expand one's own repertoire (full agreement from all participants).

**G. Decreased validity of observations and assessments**

1. Important assessments should be conducted in a supplementary face-to-face unit if specific information cannot be gathered via a video call session.
2. Clients should be informed that their clothing of choice should allow detailed observations of the body (full agreement from all participants).
3. Cameras should be positioned in a way that enables a stable image (full agreement from all participants).
4. Detailed and guided instructions on how to position the body or involved body parts in front of the camera should be given to ensure high-quality observations (full agreement from all participants).
5. The background should provide visual reference points for guiding and observing movements.
6. The observation quality of details can be improved by moving the corresponding body section closer to the camera (full agreement from all participants).
7. The observation quality of details can be improved with targeted lighting (full agreement from all participants).
8. Assessments can be adapted with the help of using objects in the client´s room during video call sessions (full agreement from all participants).
9. Increased attention should be given to small nonverbal signals of the clients.
10. Involving self-observation and self-perception of clients can supplement limited on-screen observation possibilities (full agreement from all participants).

**H. Lack of support from caregivers**

1. Ensuring that a support person is within reach can help to run a video call session smoothly (full agreement from all participants).

**I. Lack of hands-on**

1. Education about functional deficits and how to deal with them in everyday life should be emphasized in video call sessions.
2. Training self-awareness and empowering clients in their self-efficacy can be part of video call sessions to achieve lasting effects (full agreement from all participants).
3. Proven effectiveness of therapy interventions aiming at lifestyle modifications should be explained to clients (full agreement from all participants).
4. Exercises that can be performed by the clients independently should be selected (full agreement from all participants).
5. Exercises should be guided verbally in such a manner that they can be understood even if visual guidance is limited (full agreement from all participants).
6. In advance, material needed for the video call session should be communicated to the clients or relatives, and it should be prepared in a timely manner (full agreement from all participants).
7. Selected self-treatments can be taught to clients during the video call session (full agreement from all participants).
8. Clients should be given specific advice for performing self-control of guided exercises (full agreement from all participants).
9. Clients' self-awareness can be promoted by feeling movements or structures with their own hands, wherein possible misinterpretations should be considered.
10. Material required for a video call session should be communicated in advance with clear information, for example, on size, quantity, and weight.

**J. Patients or caregivers scepsis or reluctance**

1. Clients can be offered a combination of presence and video call sessions (full agreement from all participants).
2. It can be explained to skeptical clients that some therapy content is suitable for video call sessions, others not (full agreement from all participants).
3. Demonstrating video call software and discussing a possible video call session can reduce insecurities and help with decision-making.
4. A single video call session for familiarization can be offered to skeptical clients, followed by a discussion about the experience and further procedure.
5. After a few face-to-face sessions, video call sessions can be suggested again.
6. Involving relatives or the social environment can be facilitated with video call sessions.
7. The reduction of travel and increase in time flexibility can be used as an argument for using video call sessions.

**K. Decreased establishment of relationship and trust**

1. Authenticity and professionalism can be conveyed through conscious design choices and insight into the premises in the background (full agreement from all participants).
2. Optimally adjusted lighting of one's face can create a friendly atmosphere and convey mimic information well (full agreement from all participants).
3. A well-adjusted camera angle and optimal positioning in front of the screen support communication on an equal footing (full agreement from all participants).
4. Facial expressions and gestures during a video call session should be used consciously (full agreement from all participants).
5. Since real eye contact is not possible via the screen, this can be imitated by consciously looking into the camera.
6. Maintaining one's own appearance can promote authenticity in front of the camera (full agreement from all participants).
7. Conscious choice of clothing supports the professional appearance.
8. Seeing one's own camera image can be used as feedback about one’s own appearance (full agreement from all participants).
9. Framing a video call session with introductory words and a deliberate end can provide orientation (full agreement from all participants).
10. Spontaneous playful elements and humor can loosen up video call sessions and promote the relationship (full agreement from all participants).

**L. Decreased quality and effectiveness**

1. Clients who can benefit from video call sessions should be selected purposefully (full agreement from all participants).
2. The limitations of video call sessions for certain medical conditions should be clearly communicated to clients (full agreement from all participants).
3. The use of video call sessions should be evaluated regularly and adjusted if necessary.
4. Strategies to ensure whether elements learned in the session can be implemented independently should be used.
5. Video call sessions can be used to maintain a regular appointment frequency (full agreement from all participants).
6. By collecting relevant health parameters at the beginning and the end of the session, the effectiveness of treatment interventions can be evaluated.
7. At the beginning of a video call session, expectations should be gathered. At the end they should be compared with the session's content and success.
8. The role of relatives in the treatment process can be strengthened through targeted counseling during video call sessions.
9. By setting a clear focus and sequence for the video call session and individualizing the duration, one’s ability to focus can be considered (full agreement from all participants).
10. The video call session should take place during a time of the day when clients have a good ability to focus (full agreement from all participants).

**M. Increased safety risks for patients**

1. Clear exclusion and termination criteria for video call sessions for clients with safety-critical characteristics should be established and adhered to (full agreement from all participants).
2. Competent relatives can increase clients’ safety.
3. For video call sessions with children, a legal guardian should be present at the child's side (full agreement from all participants).
4. Clients or family members should be educated about making the environment as safe as possible to reduce potential hazards such as tripping, falling, or slipping (full agreement from all participants).
5. Client’s pets that may interfere with free movement in the room should be kept out of the client's room during video call sessions.
6. Exercises instructed via video call sessions should be chosen according to clients' abilities (full agreement from all participants).
7. Video call sessions should not include unfamiliar exercises that may jeopardize client safety.
8. Exercise difficulty should be increased only step by step, adapted to the client (full agreement from all participants).
9. In case of uncertainties regarding perceived health parameters, referral to specialists should be made (full agreement from all participants).
10. In psychologically tense situations, one’s own voice can serve as an instrument for calming down (full agreement from all participants).
11. The daytime of the video call session, and therefore a potentially limited suitability of certain measures, should be taken into consideration.

**N. Unsuitability of setting for telehealth**

1. A quiet room without distractions should be chosen for video call sessions (full agreement from all participants).
2. Background noise from people, pets, or household equipment should be reduced (full agreement from all participants).
3. For video call sessions, a room with good sound quality and reduced hall should be chosen (full agreement from all participants).
4. The speaker used should be tuned sufficiently loud (full agreement from all participants).
5. Balanced lighting conditions allowing good contrasts should be selected (full agreement from all participants).
6. Devices should be positioned stable to not produce blurry images (full agreement from all participants).
7. If a tablet or smartphone is used, it should be positioned at a 90-degree angle to the surface with a suitable mount (full agreement from all participants).
8. Clients should be guided in the optimal camera setup and positioning in front of it (full agreement from all participants).
9. The camera section should be chosen so that only things that one is willing to share are visible in the background (full agreement from all participants).
10. Other people in the household or work premises should be informed that a video call session is taking place to ensure privacy (full agreement from all participants).
11. It should be discussed with the clients in which area of their premises the video session will take place in order to avoid the concern of giving insight into things they want to keep private (full agreement from all participants).
12. Windows and doors should be closed.
13. High-quality microphones and headphones can improve acoustic transmission.
14. Advice should be given on how to use built-in or external microphones to ensure good transmission and avoid noise interference.
15. Doors in the camera's field of view should be avoided to prevent unwanted privacy intrusion.
16. A dedicated therapy location—for example, at a table—can promote attention and concentration.
17. Marking one or more places where the camera is well positioned facilitates a sustainably working set-up.
18. If the video call session involves movement across the room, a flexible camera setup for rapid changes should be facilitated.

**O. Increased workload**

1. Using quick-to-use treatment or exercise methods that require little pre-planning and explanation can reduce preparation time.
2. By combining preparation time for several video units, one's own working time can be used efficiently.

### Best Practices to Address Technology Barriers

In total, 20 best practices were selected by the participants to address the barriers of unstable network connections, unreliability of hardware and software, lack of technology skills, lack of privacy and IT security, and lack of technology access (A-E in Textbox 1). For optimal video call sessions, each participant should ensure a stable network connection, free from other devices and activities that might compete for bandwidth. When issues such as an unstable connection arise, switching devices, temporarily turning off the camera, or making a phone call can provide continuity and effective communication. Participants should install and test the video call software well before the session, with technical issues being addressed directly during the call or via telephone if necessary. Health care professionals should develop digital literacy for various devices and software to assist patients with technical issues. For less tech-savvy individuals, user-friendly software with direct links, telephone support, as well as on-site help from relatives, can facilitate the use of video call applications. To ensure data privacy and security, video call software must encrypt data transmission and comply with data protection laws. Informed consent should be obtained from users, and access should be restricted to invited participants. If software has not been recommended by a certification body for use in health care, it should be reviewed concerning its data processing. To optimize video call sessions, a checklist of technical requirements should be provided, and equipment availability should be confirmed beforehand. For visually intensive sessions, a high-quality large screen is recommended.

### Best Practices to Address Health Care Practice Barriers

Overall, 85 best practices addressed practice issues such as lack of telehealth knowledge and training, decreased validity of observations and assessments, lack of support from caregivers, lack of hands-on, patients or caregivers scepsis or reluctance, decreased establishment of relationship and trust, decreased quality and effectiveness, increased safety risks for patients, unsuitability of setting for telehealth, and increased workload (F-O in Textbox 1).

Reviewing current telehealth publications and internet resources to search for material to use in telehealth sessions, as well as watching software tutorials, can enhance proficiency and confidence in conducting video call sessions, complemented by practical experience and sharing knowledge with colleagues.

For comprehensive assessments in telehealth, certain procedures may need to be supplemented with in-person visits. Clients should be guided on how to prepare for video sessions with appropriate clothing, camera positioning, lighting, and background setup to enhance the quality of observations. Additionally, using objects for adapted assessments, paying attention to subtle nonverbal cues, and involving clients in self-observation can maximize the efficacy of remote evaluations. Having a support person readily available can facilitate a smooth video call session.

In video call sessions, educating clients on functional deficits and self-management in daily life is crucial, with a focus on training self-awareness and self-efficacy for sustainable outcomes. For effective video call sessions, exercises that clients can perform independently should be chosen, and clear verbal guidance for exercises with limited visual cues should be given. This can be added with the training of self-treatments, advice for self-control during exercises, and the promotion of clients' self-awareness through tactile experiences while addressing potential misinterpretations. Clear communication and preparation of materials is necessary.

To address skepticism, clients can be offered a blend of in-person and video call sessions, emphasizing the suitability of certain therapy contents for remote delivery, with demonstrations and trial sessions helping to alleviate skepticism and showcasing the convenience and flexibility that video calls offer. To convey professionalism and authenticity in video calls, one should make deliberate design and setting choices, ensure optimal lighting for a friendly atmosphere, and use a camera angle that supports equitable communication. Conscious use of facial expressions, gestures, and clothing, along with looking into the camera to simulate eye contact, enhances authenticity. Additionally, framing the session with a clear beginning and end, along with the use of humor, can improve the interaction.

Purposeful selection of clients for video call sessions is essential, with transparent communication regarding their limitations for certain conditions. Regular evaluations should be conducted to assess effectiveness and determine if expectations are being met. Strategies to ensure whether elements learned in the session can be implemented independently should be used. Sessions should be structured in a way that they have a clear focus and sequence, an appropriate duration, and are scheduled for when the client's focus can optimize the session. Incorporating relatives into the treatment process via video call sessions can provide targeted counseling. Establishing clear criteria for excluding or terminating video call sessions is vital for clients with safety-critical concerns. Competent relatives can enhance safety. Guardian presence for children, safety education, and gradual exercise progression can ensure the clients’ well-being during remote sessions.

For effective video call sessions, it is essential to choose a quiet room with as little background noise as possible, ensuring good sound quality and balanced lighting. Proper camera setup, privacy considerations, and guidance on microphone use enhance the session quality. Additionally, creating a dedicated therapy location and facilitating flexible camera setups can improve the overall experience and engagement during sessions. Efficient use of time in video sessions can be achieved by opting for quick and easy treatment methods and combining preparations for multiple video units.

## Discussion

### Principal Findings

The findings from this study, which encompassed expert interviews and a modified Delphi process, provide a best practice guideline for telehealth in health care. The key results underscore the importance of addressing the challenges of technology accessibility, enhancing observations and assessments, creating a professional setup, building relationships and trust, and ensuring privacy and IT security. Although the majority of the panelists were PTs and SLTs, their combined professional experiences—spanning areas such as neurorehabilitation and pediatric care—have facilitated a comprehensive exploration of these practices across various health care settings, thereby complementing the aims and findings of previous studies and guidelines [21-25].

The best practice guideline offers a starting point towards a unified guide to improve telehealth across various health care settings. Prior publications have established telehealth frameworks in disciplines such as occupational therapy, physiotherapy, and speech and language therapy [21-24], which partially align with our findings. A high level of agreement is seen in these existing frameworks, particularly regarding fundamental principles of telehealth delivery. However, there remain clear gaps in the current literature on how to manage these barriers through concise best practice statements [24]. Grogan-Johnson [25] discusses the challenges of maintaining engagement in virtual settings but provides limited guidance on how to address this. Our best practice guide suggests trust-building strategies, such as non-verbal communication enhancements, environmental adjustments, and interactive session structuring. Davies et al [13] outline the importance of technological proficiency for PTs but do not provide detailed practices for managing unstable networks or unreliable hardware, where our study adds explicit recommendations for overcoming technological challenges, including strategies for low-bandwidth settings, selecting robust hardware, and providing patient education on basic troubleshooting. Other studies limit their focus to specific populations or pathologies [22,25]. Cottrell and Russell [22], for instance, provide an in-depth analysis of pre-implementation considerations and practical steps specifically within the domain of musculoskeletal physiotherapy. Their work examines technological and operational challenges, offering targeted strategies to enhance the successful deployment of telehealth services. Other guidelines have delineated key technical principles health care professionals are expected to follow to ensure a smooth and secure telehealth experience [26,27], focusing on adherence to laws and regulations and the implementation of privacy and security measures. Our study extends these principles by proposing practical, applicable strategies and emphasizes dual-focused education, targeting both health care providers and patients. It includes specific methods to address skepticism and enhance digital literacy, particularly for populations unfamiliar with telehealth.

Notably, our research revealed a strong consensus on the effectiveness of specific strategies across health care settings, such as providing detailed technical guidelines, enhancing visual and audio components of telehealth sessions, and incorporating elements that foster a sense of connection and trust between health care providers and clients. These findings offer new insights into the practical implementation and adaptation of telehealth services in diverse health care contexts.

In examining the distribution of best practices identified to address various barriers, we found notable differences. “Unsuitability of setting for telehealth” yielded the largest set of best practices despite being rated as a relatively small barrier. This contrast likely arose because many aspects of the setting (eg, lighting, device positioning, noise reduction) are easily modified by health care professionals. These direct, practitioner-driven solutions help frame the barrier as “small,” in that it can be quickly addressed through practical steps. In other words, the perception of a barrier’s magnitude may reflect how empowered providers feel to remedy it: settings are simpler to adjust, whereas more systemic issues (eg, workload, limited technology access) require external interventions and may thus seem more significant.

Additionally, a significant collection of best practices has been identified to address practical implementation barriers such as “increased safety risks for patients,” “decreased validity of observations and assessments,” “lack of hands-on,” “decreased establishment of relationship and trust,” and “decreased quality and effectiveness.” These best practices provide pragmatic strategies to navigate the range of challenges presented by telehealth, ensuring a higher standard of patient care and treatment integrity. On the other side, there were barriers that received fewer best practices to address them, such as “lack of support from caregivers” and “increased workload,” both at the lower end, with 1 and 2 identified best practices. These issues likely require organizational or systemic changes that are beyond the immediate control of the individual practitioner. Similarly, “lack of technology access” and “unreliability of hardware and software” are countered by only 3 best practices each, possibly indicating a gap between the technological requirements of telehealth and the ability of health professionals to influence these parameters.

### Reflections on the Delphi Method

Our modified Delphi approach relied on 2 rounds, underpinned by preliminary interviews that generated a well-defined set of barriers and best practices. This groundwork contributed to a high level of initial consensus—83 of 108 items achieved ≥80% agreement in round 1. While we acknowledge that time constraints and difficulty finding sufficient participants limited the scope of our panel (n=9), each participant brought substantial experience: all met strict inclusion criteria (eg, minimum 2 years’ professional practice, at least 10 video-based telehealth sessions in the previous quarter), ensuring specialized and recent expertise.

We employed a 2-threshold system: items achieving ≥80% agreement in round 1 were immediately included, while items carried forward required ≥75% agreement in round 2. This tiered approach balanced efficiency and adaptability by refining practices with moderate support without burdening participants with repeated rounds. Items garnering <50% agreement were excluded after round 1, given their limited traction; however, participants could introduce revisions or propose new items if they felt critical information was overlooked. Although this may appear stringent, it enabled us to produce a focused, consensus-driven guide for reducing barriers to video call–based telehealth within the time and resource constraints of our project.

While our study does not claim to capture every possible facet of telehealth across all health care contexts, the methodology employed aligned with recommended standards.

### Limitations

This study still has several limitations that need to be acknowledged. First, the sample size was small, consisting of only 9 eligible experts in specific health professions. Our participant sample was relatively small, yet it was purposefully recruited to encompass diverse professional backgrounds and extensive telehealth experience. This approach generated a robust consensus on best practices for video call–based telehealth. Future studies with larger or more varied panels could further enhance and validate the findings, but our current methods provide a strong foundation for ongoing research and practical implementation. Second, the heterogeneity of the participants' professions may be both an advantage and a disadvantage. On the one hand, it allowed for a broader range of perspectives and strategies to be considered than if we would have focused on only one profession. On the other hand, it could also have resulted in the exclusion of profession-specific strategies that may be essential for a successful telehealth implementation, which could be studied in the future. Each profession uses telehealth differently based on their specific clinical needs and methods. For instance, PTs may focus more on movement assessments and exercise guidance, whereas SLTs might emphasize communication techniques and auditory assessments. This variation means that certain best practices identified may be more applicable to some professions than others, potentially limiting the universal applicability of the recommendations. The small number of distinct professions included in the study may have resulted in the exclusion of specialized best practices pertinent to each field. Essential strategies unique to specific clinical scenarios or patient interactions within each profession might not have been fully captured or emphasized, thereby affecting the comprehensiveness of the best practice guide for individual disciplines.

Moreover, the findings reflect the current state and must be interpreted as such. They likely are subject to change due to the continuous evolution of professional expertise in health care and ongoing technological advancements that are likely to shape the future landscape of telehealth. Furthermore, this study relied on self-reported data from the experts. While this method is commonly used in qualitative research, it is important to acknowledge that the data collected may be subject to bias or limitations in recall. Another important consideration is the length of the final best practice list. Although it represents a comprehensive set of potential solutions, its sheer number may pose challenges for day-to-day clinical application.

### Implication of Findings

The study’s findings offer valuable insights for various stakeholders within the telehealth ecosystem, promoting enhanced effectiveness, accessibility, and sustainability of video call–based telehealth services. The best practices serve as a practical guide for clinicians to optimize telehealth delivery. Implementing these strategies can improve care quality, strengthen patient-provider relationships, and increase patient satisfaction while reducing operational burdens through streamlined session management and technical troubleshooting. Using enhanced privacy measures and user-friendly technologies ensures a secure and confident telehealth experience for patients, particularly those in remote or underserved areas. Insights from this study can also inform the development of regulations and standards that promote equitable telehealth access. By addressing practical barriers and supporting measures such as technology funding and training programs, policy makers can facilitate the broader adoption and integration of telehealth into standard health care practices. Finally, incorporating these best practices into health care curricula ensures that future practitioners are equipped with the necessary digital literacy and remote communication skills. Training programs can emphasize technical competencies and patient engagement strategies specific to telehealth, preparing health care workers for modern care delivery.

### Further Research

While this Delphi study provided insights into potential solutions, it would be beneficial to evaluate the impact of these strategies on the successful implementation of telehealth in different real-world settings by exploratory and confirmatory studies. For example, while this study focused on barriers and solutions in the selected health care professions, other health care specialties and settings may face different challenges and would come up with different best practices when implementing telehealth. Evaluating these barriers and solutions in different contexts would provide a more comprehensive understanding of telehealth implementation. Moving forward, further research could investigate strategies to streamline or prioritize these recommendations, identifying which are most beneficial for specific professional groups or patient populations. Further, it would be interesting to create educational programs to see how teaching these strategies to students would increase their telehealth proficiency.

### Conclusions

This publication presents a modified Delphi study that compiles best practices for overcoming barriers in video call–based telehealth. It includes insights from various health care professionals and focuses on enhancing telehealth's quality and effectiveness. The study emphasizes technology accessibility, professional setups, and patient-centered care. Limitations include a small sample size and self-reported data, suggesting future research should assess these strategies' real-world effectiveness, explore barriers in different health care specialties, and develop educational programs to increase telehealth proficiency.

## Appendix

Multimedia Appendix 1 Interview Guideline_Translated version_English.

## Abbreviations

PT: physiotherapist
SLT: speech and language therapist

## Data Availability

The datasets generated or analyzed during this study are available from the corresponding author on reasonable request.
