# Supplementary material for: Best Practice Guide for Reducing Barriers to Video Call–Based Telehealth: Modified Delphi Study Among Health Care Professionals
Source: JMIR Hum Factors. 2025 Mar 26;12:e64079. doi: 10.2196/64079 (PMC11982760; doi:10.2196/64079)
Supplement: Multimedia Appendix 1 [file humanfactors_v12i1e64079_app1.pdf]

INTERVIEW QUESTIONS FOR THE DELPHI STUDY

A) Technical Topics

Can telehealth be carried out with individuals who do not have suitable devices?

- If yes, how do you manage to do this?

- If no, how would you define the minimum requirements?

How do you handle the issue of internet connectivity?

- Assessment

- Interruptions or delays

- Alternative strategies

What data security measures do you implement when conducting telehealth? (Encryption, data storage, etc.)

- How do you inform patients about this? (Consent form)

How do you determine that your patients have the necessary skills to use the chosen video platform and any additional equipment (camera, microphone, headset, etc.)?

- How do you support patients in this?

How do you ensure that both your own hardware/software and that of your patients is functioning properly?

- How do you support patients in using the videoconferencing software?

- What alternative procedures do you use if necessary?

B) Practice-Related Topics

How do you convince skeptical patients to use telehealth?

How do you establish and maintain a relationship and trust through telehealth?

How do you conduct qualitative observations? How do you carry out assessments/examinations?

- What should be taken into account?

Which methods do you use to compensate for the lack of hands-on (direct manual contact with patients)?

How do you ensure that your telehealth treatments are equally effective and of high quality compared to standard (in-person) therapy?

How did you acquire your knowledge regarding the use of telehealth?

- What would you recommend to others?

How do you handle the additional time requirements associated with telehealth?

How do you deal with situations where your patients do not have anyone on site to support them?

How do you identify potential risks for your patients during a telehealth session, and how do you prevent dangerous situations that you cannot personally intervene in due to physical distance?

How can an optimal setting for telehealth be created for both the patient and for yourself?

- What should be considered regarding acoustics/noise?

- What considerations apply to the available space?

- What strategies do you use concerning camera angles?

- What do you pay attention to regarding lighting?

- How can privacy be maintained?
